# Graphene Oxide in a Composite with Silver Nanoparticles Reduces the Fibroblast and Endothelial Cell Cytotoxicity of an Antibacterial Nanoplatform

**DOI:** 10.1186/s11671-019-3166-9

**Published:** 2019-10-11

**Authors:** Mateusz Wierzbicki, Sławomir Jaworski, Ewa Sawosz, Anna Jung, Grzegorz Gielerak, Henryk Jaremek, Witold Łojkowski, Bartosz Woźniak, Leszek Stobiński, Artur Małolepszy, André Chwalibog

**Affiliations:** 1 Institute of Biology, Department of Nanobiotechnology and Experiemntal Ecology, Warsaw University of Life Sciences, Ciszewskiego 8, 02-786 Warsaw, Poland; 2 Military Institute of Medicine, Szaserów 128, 04-141 Warsaw, Poland; 3 Braster S.A., Cichy Ogród 7, 05-580 Ożarów Mazowiecki, Poland; 4 Institute of High Pressure Physics of the Polish Academy of Sciences, Sokołowska 29/37, 01-142 Warsaw, Poland; 5 Faculty of Chemical and Process Engineering, Warsaw University of Technology, Waryńskiego 1, 00-645 Warsaw, Poland; 6 Department of Veterinary and Animal Sciences, University of Copenhagen, Groennegaardsvej 3, 1870 Frederiksberg, Denmark

**Keywords:** Silver nanoparticles, Graphene oxide, Antibacterial surface, Toxicity, Fibroblasts, Endothelial cells

## Abstract

Antibacterial surfaces coated with nanomaterials, including silver nanoparticles, are considered effective alternative antimicrobial agents that can be used instead of antibiotics and chemical agents. However, reports of the potential toxicity of these materials raise questions about the safety of their use in biomedical applications. The objective of this research was to reduce the human cell cytotoxicity of silver nanoparticle-coated polyurethane foils by complexing silver nanoparticles with graphene oxide. The antimicrobial activity of nanoplatforms coated with silver nanoparticles, graphene oxide and the composite of silver nanoparticles and graphene oxide was assessed with *Salmonella enteritidis*. Cytotoxicity was analysed by an analysis of the viability and morphology of human fibroblasts, human umbilical vein endothelial cells (HUVECs) and chicken embryo chorioallantoic membrane. Additionally, the synthesis level of inflammatory proteins was examined for fibroblasts cultured on different nanoplatforms. The nanoplatform coated with the silver nanoparticles and graphene oxide composite showed strongest antibacterial properties, although nanoplatforms coated with only silver nanoparticles or graphene oxide also resulted in decreased *S. enteritidis* growth. Furthermore, a nanoplatform coated with silver nanoparticles and graphene oxide composite showed limited immunological stimulation and significantly reduced cytotoxicity towards fibroblasts, HUVECs and chicken embryo chorioallantoic membrane in comparison to the nanoplatform coated only with silver nanoparticles, due to the higher stability of the nanomaterials in the nanocomposite.

## Introduction

Materials with antibacterial surfaces have been widely explored for use in medicine and the biomedical industry [1]. Nanomaterials are considered effective alternative antimicrobial agents that can be used instead of antibiotics and chemical agents [2]. Silver nanoparticles (AgNPs) are most often used for their antibacterial properties [3]. However, nanoparticles that show antimicrobial activity, including AgNPs, especially at higher concentrations, can be toxic to human cells and possibly affect human health [4, 5]. Therefore, in the biomedical industry, the application of materials with surfaces coated with nanomaterials raises questions about their safety and toxicity.

One of the possible ways to minimalise the potential toxicity of nanomaterials is to limit their mobility without changing their antimicrobial properties. Firmly attached nanomaterials used in antibacterial surfaces that do not detach from the material reduce their toxicity for human cells [6]. One of the effective methods of coating surfaces with nanoparticles is ultrasonic technology [7]. Ultrasonic waves lead to structural changes to the nanomaterials, resulting in deagglomeration or agglomeration, depending on the nanomaterial [8]. Ultrasonic technology can also be used for the synthesis of nanocomposites from different materials, including metal ions and nanoparticles [9–11]. Sonication has been used for the assembly of different nanomaterials, including the decoration of graphene oxide (GO) flakes with AgNPs and other nanoparticles [12].

The mechanism of the antibacterial activity of nanoparticles varies between the different types of nanoparticle; however, the main processes responsible for the antimicrobial properties of nanoparticles are as follows: direct interactions with the cell components and indirect processes including oxidation of cell components and disruption of oxidoreductive processes [3]. AgNP antibacterial activity results from the direct disruption of the bacterial cell membrane by AgNPs and the released Ag+ ions, inducing synthesis of reactive oxygen species (ROS), and the collapse of the plasma membrane potential, which leads to the depletion of intracellular ATP [13–15]. GO can be cytotoxic for bacterial cells due to ROS synthesis and direct cell immobilisation on the GO surface [16, 17], caused by the high adsorption capacities of GO and GO nanocomposites [18, 19].

However, the toxicity of nanoparticles has not only been observed in bacterial cells. Generally, human cells are less vulnerable to nanoparticles than bacteria due to their larger scale, and their effective repair and defence mechanisms, but cytotoxicity has been observed, especially at high concentrations. AgNP toxicity in in vitro studies occurs in concentrations of a similar order of magnitude, although they may vary substantially for more complex different biological systems or organisms [20]. The toxicity of AgNPs for multicellular organisms is often lower due to their structural and physiological differences, such as specialised cellular tissues, including epithelial cells [21]. GO biocompatibility for human cells depends on the concentration and sheet morphology. At higher concentrations, GO can lead to plasma membrane penetration and increased synthesis of ROS [22–24].

In our previous studies, we showed that nanoplatforms composed of AgNP and GO (Ag-GO) nanocomposite have a high antimicrobial efficiency towards bacteria (*Escherichia coli*, *Staphylococcus aureus* and *Staphylococcus epidermidis*) and pathogenic yeast (*Candida albicans*), which was related with the increased ROS synthesis and plasma membrane perforation [25]. Ag-GO showed higher antibacterial activity than the AgNP or GO nanoplatforms, due to the combined activity of both nanomaterials. Here, we hypothesised that polyurethane foils coated with Ag-GO nanocomposite would have lower toxicity towards fibroblasts, human umbilical vein endothelial cells (HUVECs) and an alternative in vivo model—chicken embryo chorioallantoic membrane—than foils only coated with AgNPs.

## Results

### AgNPs and GO Formed a Nanocomposite in Hydrocolloid

**Fig. 1:**
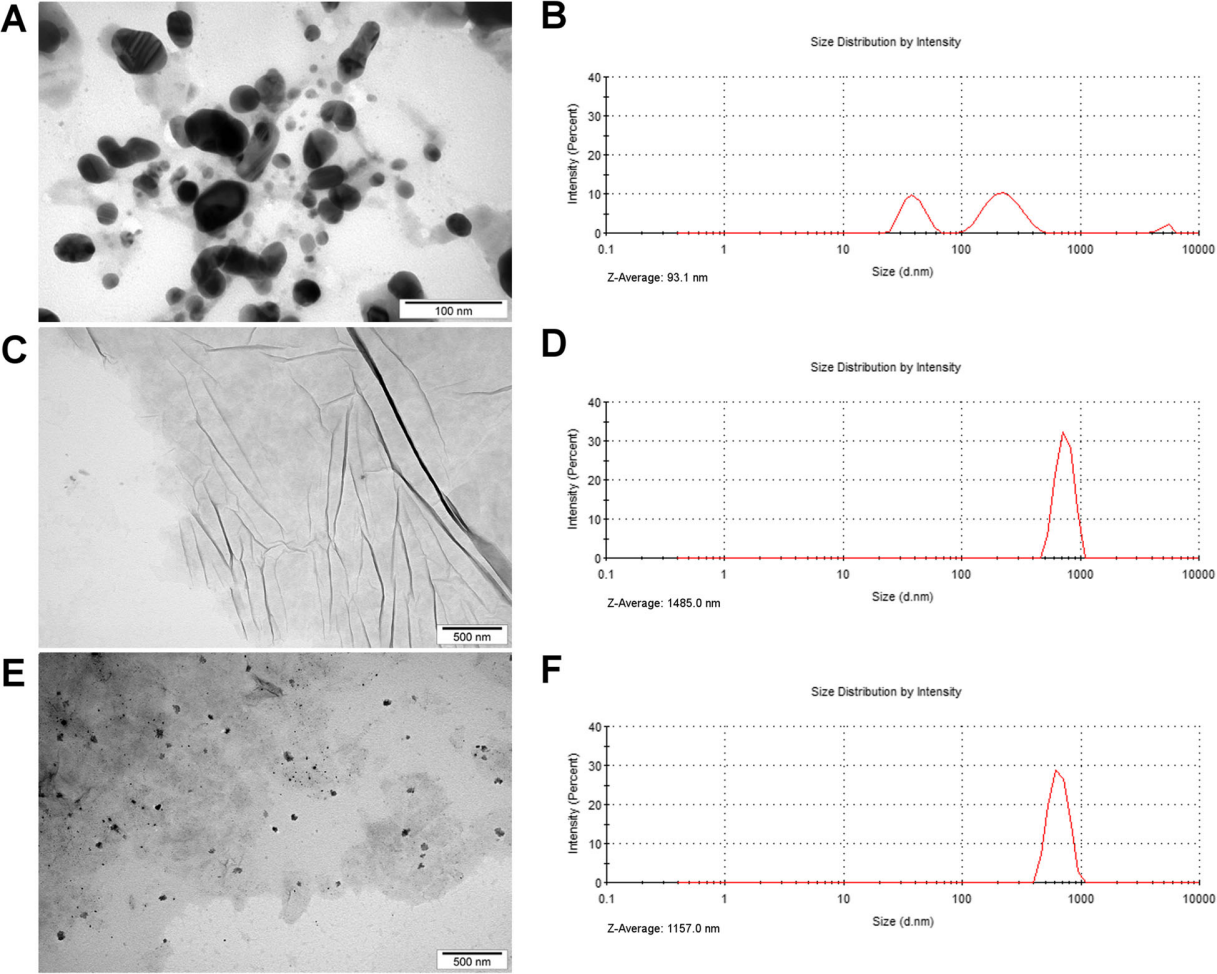
Nanoparticle morphology and size distribution. Transmission electron microscopy images of **a** silver nanoparticles, **c** graphene oxide and **e** silver nanoparticles and graphene oxide composite. Size distribution of **b** silver nanoparticles, **d** graphene oxide and **f** silver nanoparticles and graphene oxide composite

The transmission electron microscope (TEM) analysis was used to evaluate the morphology of the nanomaterials and their interactions within the Ag-GO composite (Fig. 1). AgNPs were spherical nanoparticles that had a mean size of approximately 55 nm. Additionally, TEM images showed the adhesion of silver nanoparticles to GO (Fig. 1e). These observations where further confirmed in the zeta potential analysis. The zeta potential of Ag-GO indicated that the hydrocolloid was unstable immediately after sonication, but stabilised after 24 h (zeta potential: − 15.68 and − 27.7 mV, respectively; Table 1). In contrast, the AgNP hydrocolloid was unstable both immediately after sonication and after 24 h, whereas the GO hydrocolloid was quite stable and did not change significantly after 24 h (zeta potential: − 31.11 mV and − 28.42 mV, respectively). Additionally, dynamic light scattering (DLS) analysis showed that the *Z*-average size of AgNPs was 93.1 nm, GO was 1485.0 nm and Ag-GO was 1157.0 nm. The AgNP size distribution indicated three peaks associated with agglomerate formation, while the GO and Ag-GO size distribution indicated one peak (Fig. 1b, d, f).

**Table 1:** Zeta potentials of the evaluated nanomaterials

Nanomaterial	Zeta potential after sonication [mV]	Zeta potential 24 h after sonication [mV]
Silver nanoparticles	− 2.7	− 6.5
Graphene oxide	− 31.1	− 28.4
Nanocomposite of silver nanoparticles and graphene oxide	− 15.7	− 27.7

**Fig. 2:**
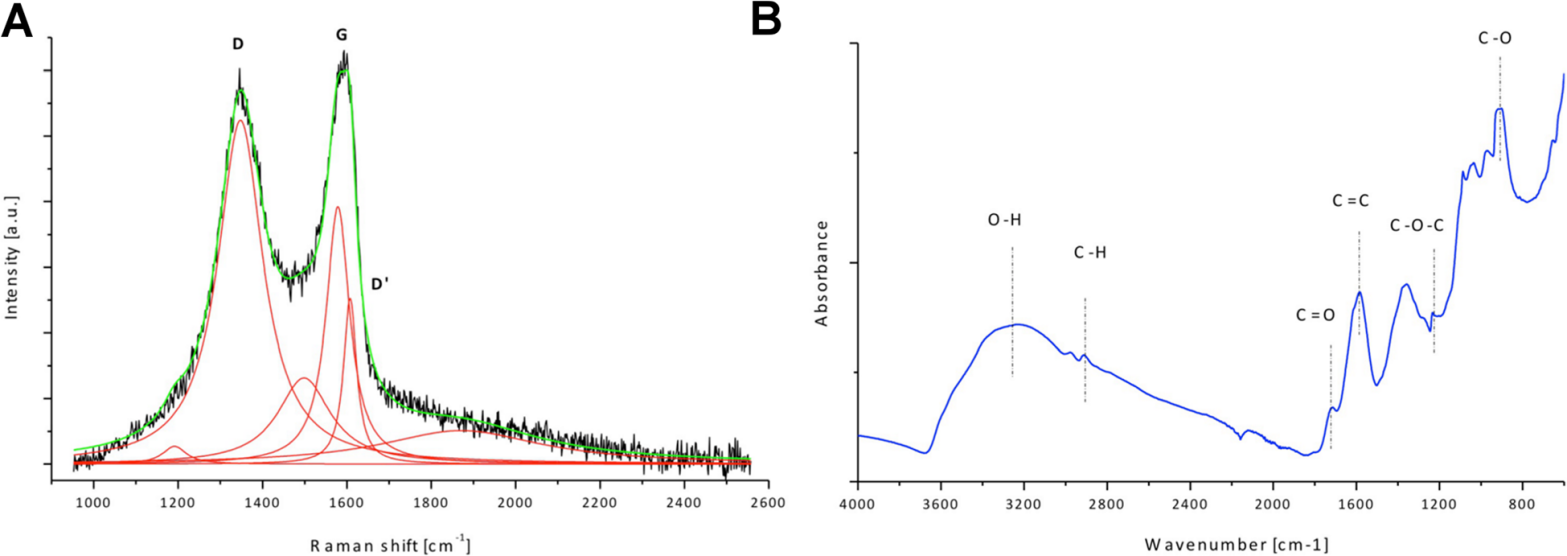
Structural feature analysis of graphene oxide. **a** Raman spectrum of graphene oxide with proposed deconvolution of the D, G and D’. **b** Fourier transform infrared spectroscopy (ATR, attenuated total reflectance) spectrum of graphene oxide with assignment of functional groups

Raman spectra and Fourier transform infrared spectroscopy (FT-IR) were used to characterise structural features of GO (Fig. 2). Figure 2a shows deconvolution of the D, G and D’ of GO. The position of the D band is 1347 cm^− 1^ and the G band 1578 cm^− 1^; the ID/IG ratio is 1.34. The FT-IR analysis revealed a broad peak observed at ~ 3500 cm^− 1^, that is assigned mainly to water and hydroxyl groups. The peak around 1600 cm^− 1^ is assigned to C=C bonds present in graphitic carbon. Other peaks observed on the FT-IR spectrum show that GO is rich in groups containing C=O bonds (mainly carboxyl groups), peaks around 1720 cm^− 1^ and 915 cm^− 1^, epoxy (C–O–C) with the visible peak around 1200 cm^− 1^, and C–H bonds (peak around 2800 cm^− 1^).

### AgNP-, GO- and Ag-GO-Coated Foils Reduced *Salmonella enteritidis* Growth

**Fig. 3:**
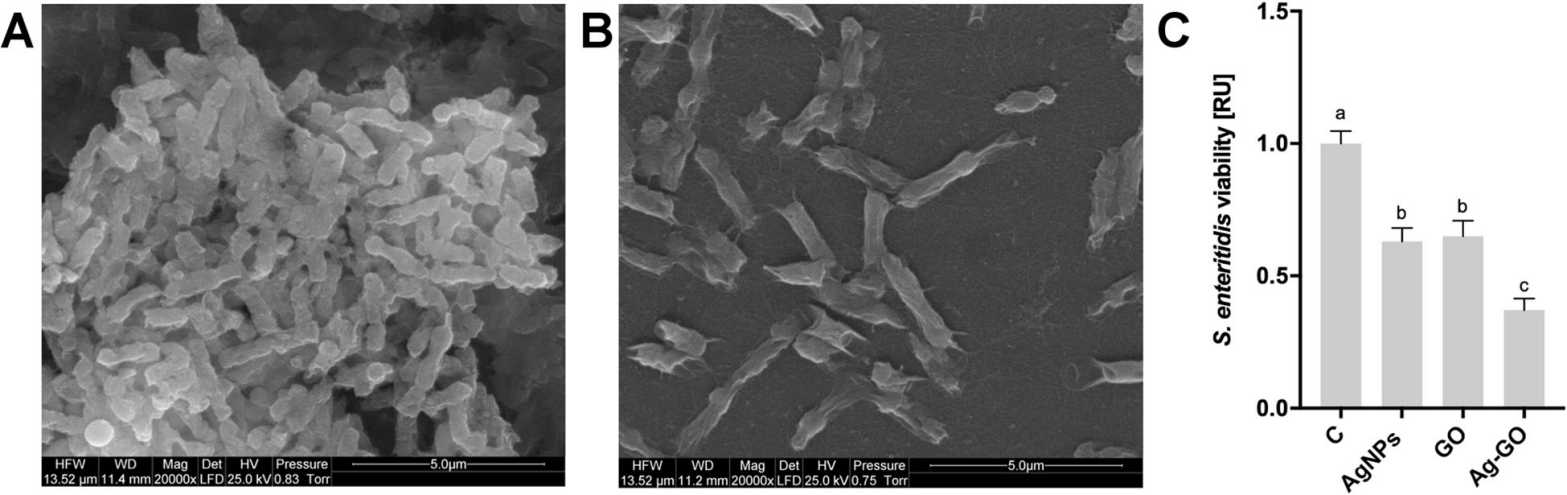
Nanoplatforms coated with silver nanoparticles and graphene oxide decreased the viability of *S. enteritidis.* Scanning electron microscope images of **a** control *S. enteritidis* bacteria and **b** *S. enteritidis* incubated on a silver nanoparticle- and graphene oxide-coated nanoplatform, after incubation at 37 °C for 24 h. **c** Viability of *S. enteritidis* after incubation on the nanoplatform for 24 h was assessed with a PrestoBlue assay. Values are expressed as mean ± standard deviation (*n* = 3, each experiment in triplicate). Statistical significance is indicated by different superscripts (one-way ANOVA; *P* < 0.05). Abbreviations: C, control group (foil without nanoparticles); AgNPs, nanoplatform coated with silver nanoparticles; GO, nanoplatform coated with graphene oxide; Ag-GO, nanoplatform coated with composite of graphene oxide and silver nanoparticles; RU, relative units

The antibacterial activity of the GO, AgNP and Ag-GO nanoplatforms was tested with *S. enteritidis*. The incubation of bacteria on foils coated with nanomaterials at 37 °C for 24 h resulted in decreased growth (Fig. 3). The strongest *S. enteritidis* growth inhibition was observed on the Ag-GO nanoplatform. However, both the AgNP and GO nanoplatforms also resulted in decreased *S. enteritidis* growth. A comparison of the scanning electron microscope (SEM) images of bacteria incubated on the Ag-GO nanoplatform to the control group showed a reduced number of *S. enteritidis* cells. Additionally, bacteria were adhered to the nanoplatforms and showed morphological changes, indicating the disruption of their cell membrane.

### AgNP Toxicity Is Inhibited by GO in an Ag-GO Composite-Coated Nanoplatform

**Fig. 4:**
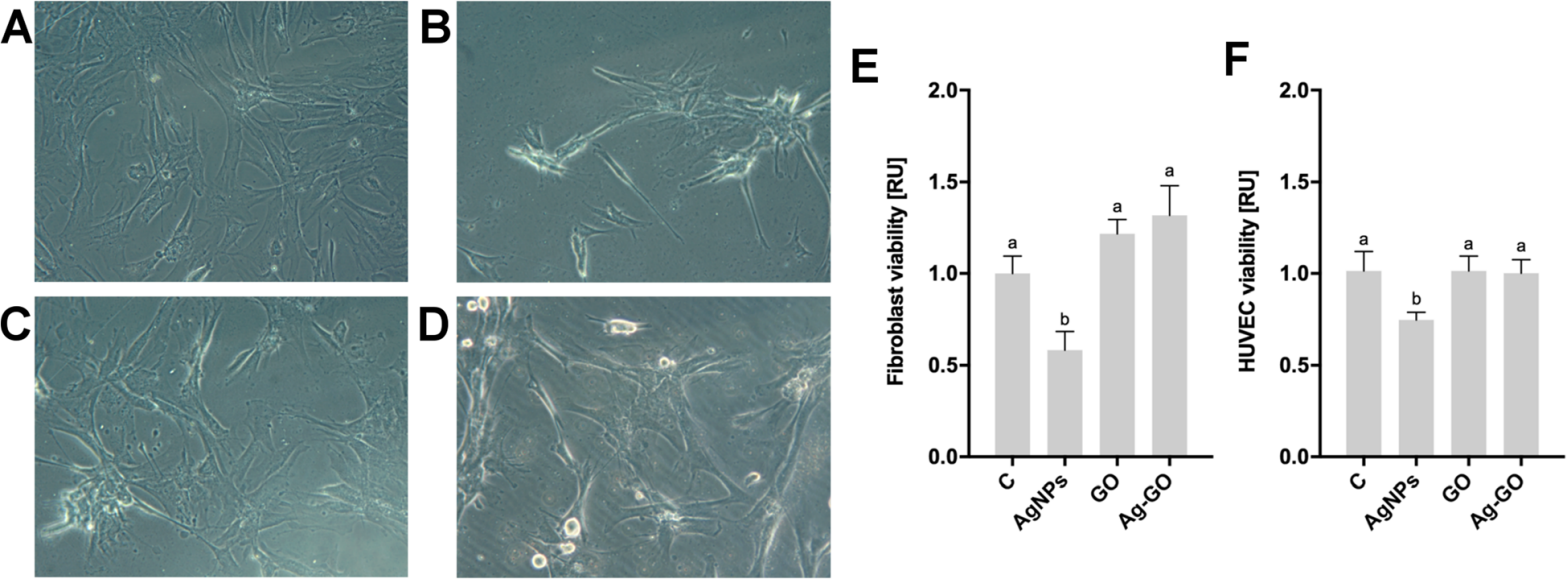
Nanoplatforms coated with graphene oxide decreased the cytotoxicity of silver nanoparticles**.** Morphology of fibroblasts cultured on **a** non-coated nanoplatforms, **b** silver nanoparticle-coated nanoplatforms, **c** graphene oxide-coated nanoplatforms, **d** silver nanoparticles and graphene oxide composite-coated nanoplatforms. Morphology was assessed by light microscopy using phase contrast with × 200 magnification. Fibroblast (**e**) and HUVEC (**f**) viability after 24 h of incubation on the nanoplatforms was determined using a PrestoBlue assay. Values are expressed as mean ± standard deviation (*n* = 3, each experiment in triplicate). Statistical significance is indicated by different superscripts (one-way ANOVA; *P* < 0.05). Abbreviations: HUVECs, human umbilical vein endothelial cells; C, control group (foil without nanoparticles); AgNPs, nanoplatform coated with silver nanoparticles; GO, nanoplatform coated with graphene oxide; Ag-GO, nanoplatform coated with a composite of graphene oxide and silver nanoparticles; RU, relative units

The toxicity of nanoplatforms was investigated by the direct incubation of fibroblasts and HUVECs for 24 h on nanoplatforms and uncoated foils (Fig. 4). There were significant differences between the viability of both fibroblasts and HUVECs on the different nanoplatforms (*P* = 0.0003 and *P* = 0.0156, accordingly). GO nanoplatforms did not change the viability of fibroblasts, compared to the viability of cells incubated on uncoated foils. Similarly, there was no significant impact of GO on the viability of HUVECs. However, coating with AgNPs resulted in a 40–50% decrease of the viability of both fibroblasts and HUVECs. The cell viability of fibroblasts and HUVECs was not changed when they were incubated on nanoplatforms coated with Ag-GO nanocomposite, showing the inhibition of AgNP toxicity. Cell morphology on uncoated foils showed the typical morphology of fibroblasts grown in 2D culture conditions (Fig. 4a). Cells incubated on AgNP-coated foil showed an intensive aggregation of cells. Cell morphology on the GO- and Ag-GO-coated nanoplatforms showed a reduction of agglomeration tendencies and cell spreading.

**Fig. 5:**
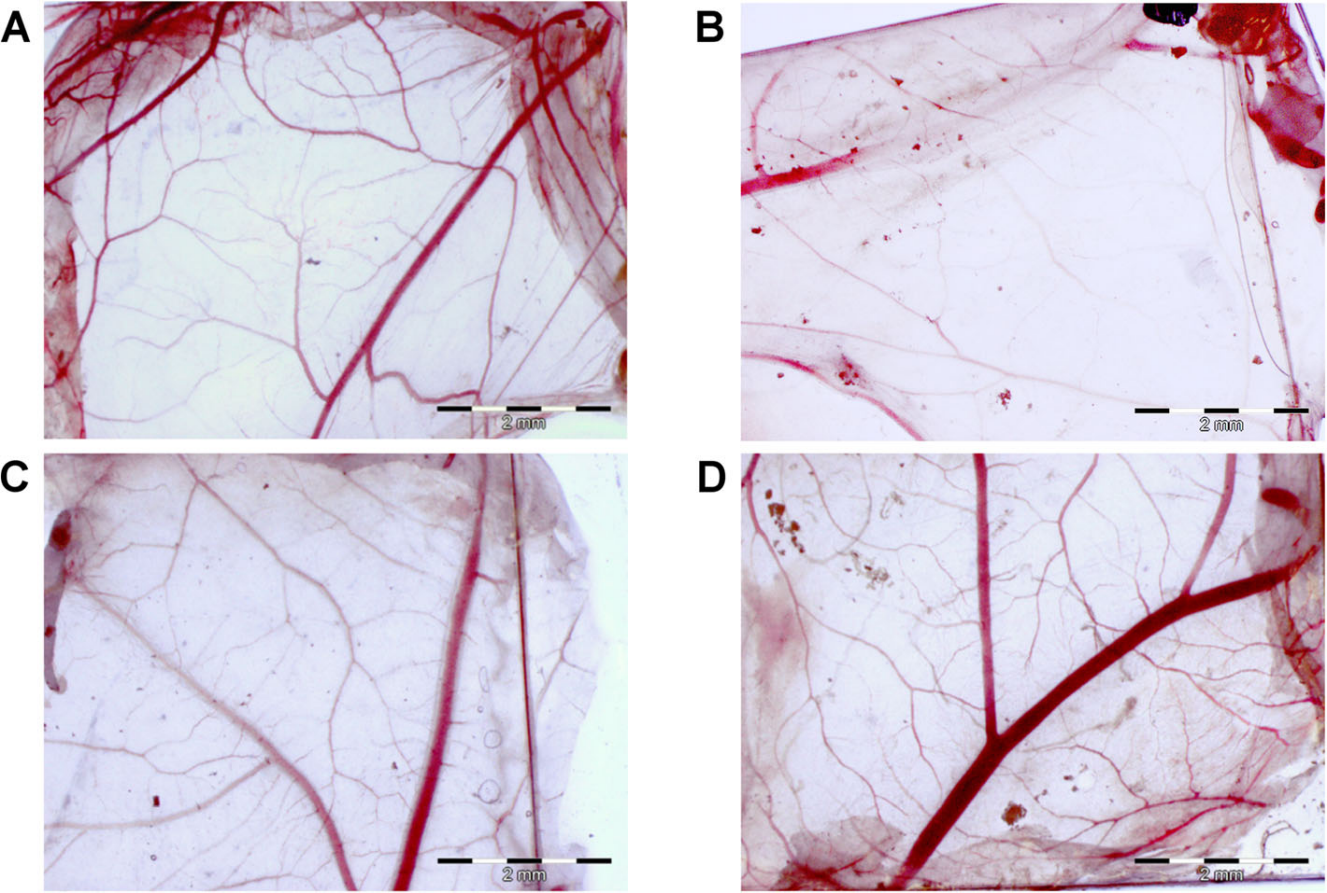
Graphene oxide decreased the morphological changes of the chorioallantoic membrane caused by silver nanoparticles. The morphology of the chicken embryo chorioallantoic membrane after 48 h of incubation with the nanoplatforms. **a** Control group (foil without nanoparticles), **b** nanoplatform coated with silver nanoparticles, **c** nanoplatform coated with graphene oxide, **d** nanoplatform coated with composite of graphene oxide and silver nanoparticles

Nanoplatform toxicity was also evaluated using a chicken embryo chorioallantoic membrane (Fig. 5). Nanoplatforms were incubated directly on a chorioallantoic membrane, and its morphology at the place of contact was examined after 48 h. AgNPs caused morphological changes to the chorioallantoic membrane, whereas in the case of the GO and Ag-GO nanoplatforms, the morphology was comparable to that of the control group (Fig. 5b). The chorioallantoic membrane, after incubation on the AgNP nanoplatform, showed a decreased number of capillary vessels, suggesting direct toxicity to the endothelial and mesenchyme cells.

### AgNPs Decreased the Release of Interleukins 6 and 8

**Fig. 6:**
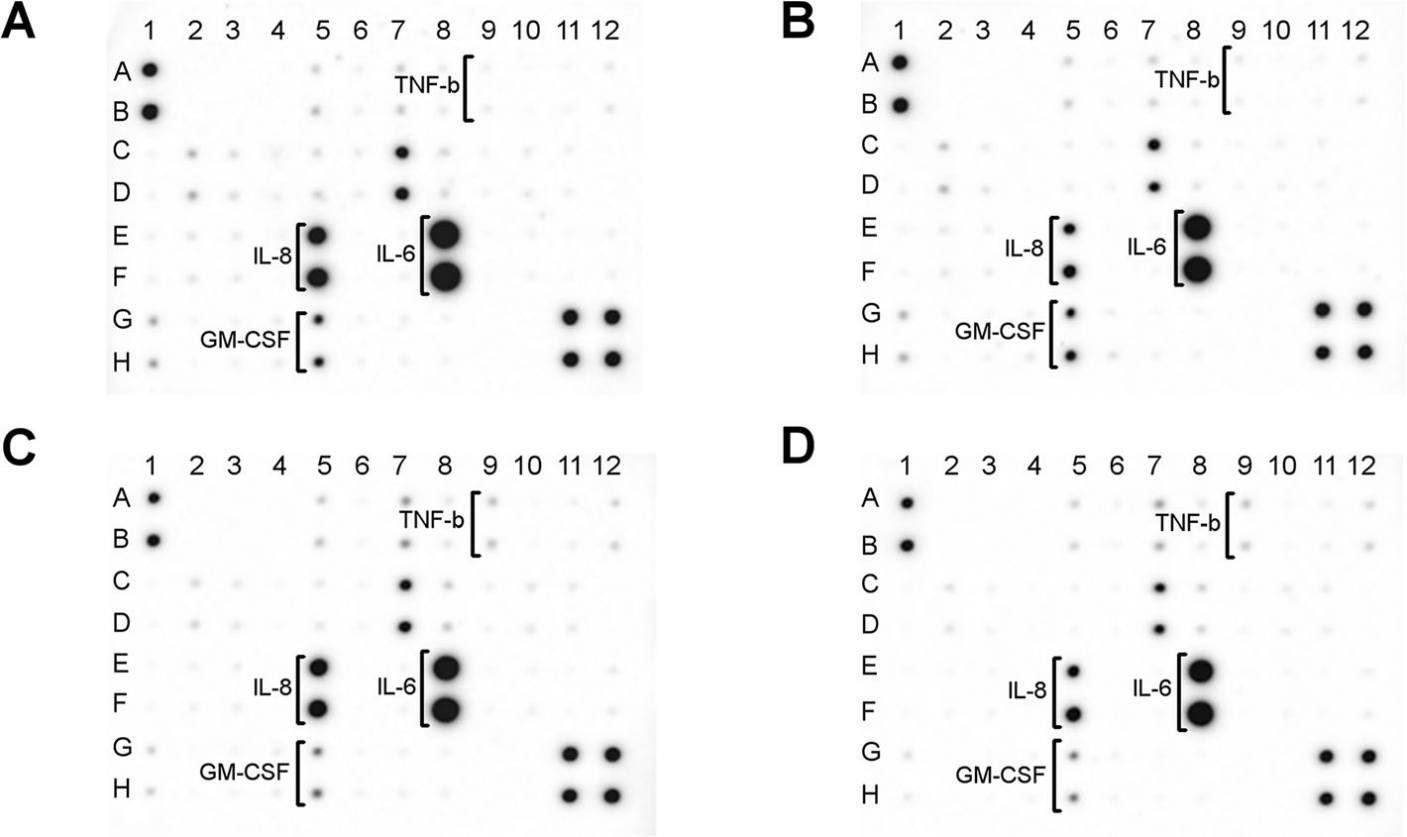
Antibody array analysis of the inflammatory cytokine release of fibroblasts after 24 h of incubation. **a** Control group (foil without nanoparticles), **b** nanoplatform coated with silver nanoparticles, **c** nanoplatform coated with graphene oxide, **d** Ag-GO nanoplatform coated with composite of graphene oxide and silver nanoparticles. The AgNP and Ag-GO nanoplatforms decreased the release level of IL-8 (dots: E5, F5). Both the GO and Ag-GO nanoplatforms decreased the synthesis of GM-CSF (dots: G5, H5). Additionally, the GO and Ag-GO nanoplatforms led to the increased synthesis of TNF-β (dots: A9, B9). The AgNP, GO and Ag-GO nanoplatforms decreased the release level of IL-6 (dots: E8, F8). A full array map is available in Additional file 1

An antibody array was used to analyse the cell media content of 40 inflammatory proteins synthetised by fibroblasts (Fig. 6). The main inflammatory proteins released by fibroblasts were interleukin 8 (IL-8; Fig. 6, dots: E5, F5) and interleukin 6 (IL-6; Fig. 6, dots: E8, F8). The AgNP and Ag-GO nanoplatforms significantly decreased the release level of IL-8, whereas the GO nanoplatform did not have such an effect. Additionally, both the GO and Ag-GO nanoplatforms decreased the release level of granulocyte-macrophage colony-stimulating factor (GM-CSF; Fig. 6, dots: G5, H5). The GO and Ag-GO nanoplatforms also led to the increased release level of tumour necrosis factor beta (TNF-β; Fig. 6, dots: A9, B9). Interestingly, AgNP, GO and Ag-GO nanoplatforms significantly decreased the release level of IL-6. The level of release of the other analysed proteins was not changed. An array map with a list of all the analysed cytokines is included in Additional file 1: Figure S1.

## Discussion

In biomedical applications, the safety of nanomaterials used in antimicrobial materials is as important as their efficiency in killing bacteria. In this study, we showed that coating materials with GO can efficiently decrease the toxicity of nanomaterials. Polyurethane foil coated with AgNPs and GO (Ag-GO) not only increased their antimicrobial properties, but also decreased their toxicity in human cells.

Raman spectroscopy was used to analyse structural features of graphene oxide. The G band in the Raman spectra corresponds to sp^2^ hybridised carbon-based material [26]. The D peak is related to defect or lattice disorder due to the binding of oxygen-functional group [27]. The intensity of the D band is associated with the size of the sp^2^ in-plane domains [26]. The additional bands D’ arise from the defects present in the graphitic structure of the carbon material. ID/IG ratio (calculated from the intensity of D and G bands) can be used to characterise the disorder of the graphitic structure in carbon materials. As demonstrated, GO has a highly disordered structure due to many functional groups in the structure formed during the oxidation of graphite powder [28].

The FT-IR spectrum of graphene oxide collected in the ATR mode revealed that GO has a lot of functional groups present in the structure, including carboxyl and epoxy groups, peaks around 1720 cm^− 1^ and 915 cm^− 1^, epoxy (C–O–C) with the visible peak around 1200 cm^− 1^, and C–H bonds (peak around 2800 cm^− 1^). The FT-IR analysis is in good agreement with XPS measurements performed for GO where also hydroxyl, carboxyl, epoxy and carbonyl groups were identified [29].

The coating of nanomaterials was performed with ultrasonic technologies, which have been confirmed to be an effective method of coating various materials with antibacterial and fungicidal substances, including AgNPs [30, 31]. Ultrasonic waves utilise cavitation phenomena by generating and collapsing cavitation bubbles, producing high energy and pressure [32]. Nanomaterials accelerated to high velocities collide with the coated material and are deposited on the surface [33]. However, the effectivity of nanomaterial deposition can be increased not only by using a proper coating method, but also by making composites with nanomaterials that can be more easily attached to the surface. GO is a favourable nanomaterial for creating stable composites with both different nanomaterials and surfaces. Due to its unique structure, with carbon atoms in a hexagonal pattern with numerous oxygen-containing functional groups in close proximity, including carboxyl and hydroxyl groups, GO is prone to form covalent bonds or electrostatic interactions [34]. Usually, GO-nanoparticle composites are synthesised by the attachment of metal ions or metal nanoparticles to GO surfaces through electrostatic or covalent interactions. Additionally, the reduction of metal ions and/or GO is performed to form covalent bonds [35]. Ag-GO composites have been made using ultrasonication by the in situ reduction of Ag^+^ [36, 37], as well by the deposition of AgNPs [12]. In our previous report, we showed that ultrasonic methods can be used to synthesise Ag-GO-coated nanoplatforms on polyurethane foils [25]. However, sonication not only led to coating polyurethane foils with nanomaterials, but also to the formation of an Ag-GO composite. The formation of the Ag-GO composite, even before coating the foils, could have result in the greater stability of AgNPs after coating.

In our studies, fibroblasts and HUVECs were used for cytotoxicity studies followed by analysis of chicken embryo chorioallantoic membrane. Skin fibroblasts are considered as a good model for skin irritation studies compared to in vivo analysis [38], whereas endothelial cells, including HUVEC cytotoxicity, are often studied due to probable direct contact of nanoparticles in biomedical applications and sensitivity of these cells to nanoparticles [39, 40]. Chicken embryo chorioallantoic membrane is an alternative in vivo model to rodent models for various toxicology studies including material toxicology and acute toxicological studies [41, 42].

Fibroblasts and HUVECs had higher viability when grown on Ag-GO than AgNP-coated nanoplatforms. Additionally, the AgNP nanoplatform caused morphological changes in the chorioallantoic membrane, whereas in the case of the GO and Ag-GO nanoplatforms, cell morphology was comparable to the control group. The decrease of toxicity on the Ag-GO nanoplatform could result from the combined effects of a higher stability of AgNPs in the complex with GO and a better deposition of AgNPs in the nanoplatform. The toxicity of animal cells is often more severe after nanoparticles have entered the cell by direct penetration or endocytosis [43]. Nanoparticle endocytosis is size and shape dependent. Bigger particles and composites are taken up to a lesser extent than particles that are approximately 45 nm in size [44]. The most noticeable relation between endocytosis and shape or size of nanomaterials is characteristic for carbon wall nanotubes. Nanotubes with length less than 1 μm effectively penetrate plasma membrane through direct diffusion, whereas phagocytosis or endocytosis pathways internalise longer nanotubes and agglomerates [45]. Recently, the Ag-GO nanocomposite was shown to be internalised by J774 macrophages, approximately 60% less than AgNPs. However, because Ag-GO induced more ROS, the overall toxicity for the cells was higher [46]. Additionally, kinetic analysis of shape-dependent internalisation of nanoparticles shows that spherical sized nanoparticles are generally internalised much faster than flat particles [47]. Furthermore, the toxicity of nanoparticles for human cells is usually size dependent, in which smaller particles show stronger cytotoxic properties. In studies on the size-dependent cytotoxicity of AgNPs for RAW, 264.7 macrophages and L929 fibroblast nanoparticles had lower viability after treatment with 20-nm AgNPs than after treatment with larger nanoparticles (80, 113 nm) [13]. Therefore, the increased size of Ag-GO composites and the decreased ability of cells to uptake nanoparticles resulting from stable deposition to the surface could be the reason for the observed higher viability of both HUVECs and fibroblasts cultured on the Ag-GO nanoplatform.

The AgNP and Ag-GO nanoplatforms significantly decreased the release level of IL-8 by fibroblasts. The GO and Ag-GO nanoplatforms led to an increased release of TNF-β. Additionally, AgNP, GO and Ag-GO nanoplatforms decreased the release of IL-6. Interestingly, changes in the synthesis of proinflammatory proteins by fibroblasts were related to the incubation of cells on nanoplatforms coated with AgNPs or GO. The synthesis levels of cells incubated on Ag-GO did not differ from those incubated on nanoplatforms coated with only one of these nanomaterials, suggesting that biological activity did not change after the synthesis of the composite. Fibroblasts are important in inflammatory and remodelling processes by initiating inflammatory responses and precipitating in the switch from acute inflammation to tissue repair [48, 49]. Therefore, the analysis of fibroblast secretions of inflammatory cytokines is important to predict the immunological response to nanoplatforms. Both IL-6 and IL-8 are one of the key inflammatory cytokines that, after synthesis by fibroblasts, leads to the activation of the immunological response [50, 51]. The human epidermal keratinocyte synthesis level of IL-6 decreases after treatment with AgNPs [52]. Similarly, the inhibition of IL-6 release by AgNPs was demonstrated in Jurcat cells and involves the MAPK pathway. AgNPs also decrease the synthesis levels of tumour necrosis factor alpha (TNF-α) [53]. TNF-α and, very similar in structure and function, TNF-β are inflammatory cytokines that are important during the acute inflammation phase. Although immune cells are mainly responsible for the release of those proteins during the acute phase of inflammation, fibroblasts and different cells are involved in the synthesis of inflammatory cytokines during the early process of wound healing [54]. Activity to induce TNF-α after treatment with GO was demonstrated using RAW264.7 macrophages [55], which suggests immunological stimulation. However, in our studies, the release levels of most of the analysed proinflammatory proteins were not changed after the cells were cultured on the GO and Ag-GO nanoplatforms. Therefore, these analyses suggest that both the GO and Ag-GO nanoplatforms possess good biocompatibility and should not lead to strong immunological reactions.

## Conclusions

In conclusion, the presented results show that nanoplatforms coated with an Ag-GO composite have showed stronger growth inhibition of *S. enteritidis* than AgNP- and GO-coated nanoplatforms. Moreover Ag-GO composite significantly reduced cytotoxicity towards fibroblasts, HUVECs and chicken embryo chorioallantoic membrane, in comparison to nanoplatforms coated with AgNPs. The cell viability of fibroblasts and HUVECs was not changed when they were incubated on nanoplatforms coated with Ag-GO nanocomposite, showing the inhibition of AgNP toxicity. These results, together with low immunological stimulation, suggest that the GO could be used for reduction of cytotoxicity of different nanomaterials in nanocomposites. Furthermore, the results suggest that the Ag-GO nanoplatform could be considered for use in biomedical applications. However, additional studies are needed to evaluate Ag-GO nanoplatform for specific applications, including wound dressings.

## Materials and Methods

### Preparation and Characterisation of Nanoplatforms Coated with Nanomaterials

Nanoplatforms made from nanoparticle-coated polyurethane foils were prepared as previously described [25]. Square-shaped polyurethane foils (15 × 15 mm, 0.05 mm thick) were covered with suspensions of AgNPs (HydroSilver1000, Amepox, Łódź, Poland) synthetised by chemical reduction reaction in the presence of polyvinyl alcohol developed by Amepox and/or GO synthetised by modified Hummers’ method. Ten grams of graphite powder was mixed with 230 ml of concentrated sulphuric acid (98%) (Sigma-Aldrich Co., St. Louis, MO, USA) at a temperature below 10 °C. Subsequently, 4.7 g of sodium nitrate (Sigma-Aldrich) and 30 g of potassium permanganate (Sigma-Aldrich) were added to the graphite mixture, while keeping the temperature below 10 °C. Then, the mixture was heated to 30 °C and stirred for 2 h. Subsequently, 100 ml of water was added and the mixture was treated with 10 ml of hydrogen peroxide. GO was purified by filtration and washed with deionised water until the pH of the filtrate reached 6.5. Suspensions of GO, AgNPs and the composite of AgNPs and GO (Ag-GO) were prepared in deionised water. During coating, the concentrations of nanomaterials were as follows: GO, 200 mg/l; AgNPs, 100 mg/l; Ag-GO, 200 mg/l; and AgNPs, 100 mg/l. Nanoparticle coating was performed using an ultrasonic horn (Ti horn, Ø13 mm, 60% efficiency, 20 kHz; Sonics & Materials, Inc., Newtown, CT, USA) at a temperature of 30 ± 1 °C. The covered samples were flushed in deionised water and dried in sterile conditions. Nanoplatform characterisation with a scanning electron microscope (SEM), atomic force microscope (AFM) and lateral force microscope (LFM) has been previously reported, showing nanoplatforms almost entirely covered with nanomaterials [25].

The nanomaterials used to obtain the nanoplatforms were imaged using a transmission electron microscope (TEM). TEM images were acquired using a JEM-1220 microscope (JEOL, Tokyo, Japan) at 80 kV with a Morada 11-megapixel camera (Olympus Corporation, Tokyo, Japan). Samples were prepared by placing droplets of hydrocolloids onto formvar-coated copper grids (Agar Scientific, Stansted, UK), which were allowed to air-dry before observations.

Raman spectra were collected using a Renishaw inVia spectrometer with a 532-nm laser source (Wotton-under-Edge, UK). To avoid heating of the sample, the laser power was kept low (0.3 mW, calibrated on the sample). The Raman mapping mode was used with a scan area of approximately 10 × 10 μm, containing 25 spectra). Each spectrum consisting of two main bands, a G band (~ 1578 cm^− 1^) and D band (~ 1347 cm^− 1^), was fit using Lorentzian line shape. FT-IR measurements were performed using a Nicolet iS10 spectrometer (Thermo Fisher Scientific, Waltham, USA) in attenuated total reflectance mode on a diamond crystal. Graphene oxide suspension was dried on the polyethylene surface at room temperature to create GO thin foil. The spectrum was collected in the range 400–4000 cm^− 1^.

Zeta potential measurements of GO (20 mg/l), AgNPs (10 mg/l) and Ag-GO (GO 20 mg/l and AgNPs 10 mg/l) were carried out with a Nano-ZS90 Zetasizer (Malvern Instruments, Malvern, UK) at 25 °C, using the Smoluchowski approximation. Nanomaterials were sonicated for 30 min and zeta potential was immediately measured. Subsequently, nanomaterials were left for 24 h at room temperature and the zeta potential was measured again. Each measurement was repeated at least seven times after 60 s of stabilisation at 25 °C.

The hydrodynamic diameter of nanoparticles in water and their size distribution were measured with dynamic light scattering (DLS) using a Nano-ZS90 Zetasizer (Malvern). Similar to for the zeta potential analysis, GO (20 mg/l), AgNPs (10 mg/l) and Ag-GO (GO 20 mg/l and AgNPs 10 mg/l) were sonicated for 30 min and left for 24 h at room temperature. Each sample was measured at least seven times at 25 °C.

### Bacterial Cultivation

*Salmonella enteritidis subspecies enterica serovar Enteritidis* (ATCC 13076) was obtained from LGC Standards (Łomianki, Poland). The bacteria were grown on tryptic soy agar (Merck Millipore, Darmstadt, Germany). The bacteria, grown on agar plates, were harvested by gently washing the plates with sterile distilled saline solution. To calculate the number of bacteria in the cell suspension, the optical density of the suspensions at 600 nm (OD600) was measured using a spectrophotometer (Helios Epsilon, Unicam, Milwaukee, WI, USA). A calibration curve was prepared by performing serial tenfold dilutions of bacterial suspensions of a known optical density, up to 10^− 5^. After 24 h of incubation at 37 °C, the number of formed colonies was enumerated and the number of colony-forming units (CFU) of the original bacterial suspension was calculated.

### Bacteria Viability Assay

Viability was evaluated using a PrestoBlue Cell Viability Assay (Thermo Fisher Scientific). Bacteria were cultured onto foils coated with GO, AgNPs and Ag-GO, located on inserts inserted into six-well plates (200 μl MH broth with 5 × 10^3^ CFU per foil) and incubated for 24 h. Subsequently, 90 μl of each sample was transferred to 96-well plates and 10 μl of PrestoBlue reagent was added to each well and incubated for an additional 2 h at 37 °C. The optical density of each well was recorded at 570 nm using a microplate reader (Infinite M200, Tecan, Durham, NC, USA). Bacteria viability was expressed as the relative value after substitution of the absorbance from the blank samples. Experiments were repeated three times.

### Scanning Electron Microscopy Analysis

Bacteria were incubated on foils with Ag-GO and a sterile cover glass. Bacteria cultures (100 μl, 10^6^ CFU/ml) were incubated on foils and a cover glass for 24 h at 37 °C. All samples were dried and covered with gold. Cells were fixed with 2.5% glutaraldehyde in phosphate-buffered saline (PBS, pH 7.2) and contrasted with 1% osmium tetroxide (Sigma-Aldrich) and 1% carbohydrazide (Sigma-Aldrich). Subsequently, cells were dehydrated in increasing concentrations of hexylene glycol (Sigma-Aldrich). Drying was performed using a Polaron CPD 7501 critical point dryer (Quorum Technologies, Laughton, UK). Finally, the samples were imaged with a SEM (FEI Quanta 200, Tokyo, Japan) at an acceleration voltage of 15 kV.

### Human Cell Lines

Human umbilical vein endothelial cells (HUVECs; catalogue number: C0035C) and human fibroblasts (catalogue number: C0135C) were obtained from Thermo Fisher Scientific. HUVECs were maintained on low-serum Medium 200 basal media supplemented with Large Vessel Endothelial Supplement (Thermo Fisher Scientific) and 1% penicillin/streptomycin (Thermo Fisher Scientific), whereas fibroblasts were cultured in low-serum Medium 106 (Thermo Fisher Scientific) supplemented with Low Serum Growth Supplement (Thermo Fisher Scientific) and 1× penicillin/streptomycin (Thermo Fisher Scientific). Cells were maintained at 37 °C in a humidified atmosphere of 5% CO_2_/95% air.

To analyse biological interactions, the nanoplatforms were put into six-well plates. After detachment from the cell culture flask, HUVECs or fibroblasts were placed directly on the nanoplatform with 100 μl of growth media. To avoid the media drying during incubations, plates were kept in humidity chambers.

### Analysis of Nanoplatform Toxicity to HUVECs and Fibroblasts

To analyse HUVEC and fibroblast viability on the nanoplatforms, cells were cultured in the droplet directly on the nanoplatforms or uncoated foil (1 × 10^4^ cells in 100 μl growth media). After 24 h of incubation, cell viability was analysed using a PrestoBlue assay (Thermo Fisher Scientific). PrestoBlue reagent was incubated with assessed cells for 2 h in a cell culture incubator. Subsequently, 50 μl of growth media with PrestoBlue reagent was transferred to a 96-well plate where fluorescence (excitation *λ* = 560 nm, emission *λ* = 590 nm) was analysed using a Tecan Infinite 200 microplate reader (Tecan, Durham, USA). Cell viability was expressed as the relative value after substitution of the fluorescence from blank samples. Experiments were repeated three times.

Fibroblast morphology was observed using an inverted optical microscope (Olympus Corporation) using phase contrast. Fibroblasts were seeded in 35-mm diameter Petri dishes directly on the nanoplatforms (1 × 10^4^ cells in 100 μl growth media). Images were taken after 24 h of incubation.

### Chorioallantoic Membrane Assay

Fertilised eggs from Ross 308 hens were obtained from a certified hatchery and kept for 4 days at 12 °C. The eggs were cleaned, sterilised with UVC light and divided into four groups (4 × 20 eggs). Embryos were incubated at standard conditions (temperature 37 °C, humidity 60% and turned once per hour). At 8 days of embryonic development, small holes (1 cm^2^) were made in the shell above the air space, the inner membrane was gently striped off and the nanoplatforms were placed on the chicken embryo chorioallantoic membrane. Subsequently, chicken embryos were incubated for the next 48 h, when nanoplatforms were cut out with the chorioallantoic membrane that was directly below the nanoplatform. The chorioallantoic membrane on the nanoplatforms was imaged using a stereoscopic microscope (SZX10, Olympus Corporation).

### Antibody Array Analysis

An analysis of inflammation cytokines in fibroblast growth medium was performed using an antibody array (Abcam, Cambridge, UK; catalogue number ab134003). Fibroblast cells (1 × 10^4^) were incubated on nanoplatforms coated with AgNPs, GO, Ag-GO and uncoated foil with 100 μl of media. After 24 h, 80 μl of growth medium was collected. For each experimental group, the growth medium from six foils was used for analysis. Pooled growth medium from the six experiments was centrifuged (1600 rpm for 5 min), and 500 μl of growth media was diluted in 500 μl of PBS. Therefore, 1 ml of diluted growth media was used per each analysed membrane. The assay was performed in accordance with the manufacturer’s instructions. Diluted growth media was incubated with the membranes for 24 h at 4 °C. Subsequently, antibodies conjugated with biotins were added and incubated for the next 24 h at 4 °C. In the next step, the membranes were incubated with streptavidin conjugated with horseradish peroxidase for 2 h at room temperature. Membranes were visualised after the addition of the provided horseradish peroxidase substrate using a ChemiDoc imaging system (Bio-Rad, Hercules, USA).

### Statistical Analysis

Data were analysed using one-way analysis of variance with GraphPad Prism 8 (GraphPad Software, San Diego, CA, USA). Differences between groups were tested with Tukey’s HSD post hoc tests. Results are shown as means with standard deviations. Differences at *P* < 0.05 were considered significant.

## Supplementary information

**Additional file 1:** Figure S1. Antibody array map.

## Data Availability

The datasets used and/or analysed during the current study are available from the corresponding author on reasonable request.

## Abbreviations

Ag-GO: Composite of silver nanoparticles and graphene oxide
AgNPs: Silver nanoparticles
CFU: Colony-forming units
DLS: Dynamic light scattering
FT-IR: Fourier transform infrared spectroscopy
GM-CSF: Granulocyte-macrophage colony-stimulating factor
GO: Graphene oxide
HUVECs: Human umbilical vein endothelial cells
IL-6: Interleukin 6
IL-8: Interleukin 8
SEM: Scanning electron microscope
TEM: Transmission electron microscope
TNF-α: Tumour necrosis factor alpha
TNF-β: Tumour necrosis factor beta
