# Supplementary material for: Graphene Oxide in a Composite with Silver Nanoparticles Reduces the Fibroblast and Endothelial Cell Cytotoxicity of an Antibacterial Nanoplatform
Source: Nanoscale Res Lett. 2019 Oct 11;14:320. doi: 10.1186/s11671-019-3166-9 (PMC6787127; doi:10.1186/s11671-019-3166-9)
Supplement: Supplementary file 1 — Additional file 1: Figure S1. Antibody array map. [file 11671_2019_3166_MOESM1_ESM.docx]

**Additional file**

**Graphene oxide in a composite with silver nanoparticles reduces the fibroblast and endothelial cell cytotoxicity of an antibacterial nanoplatform**

*Mateusz Wierzbicki^1^***, Sławomir Jaworski^1^, Ewa Sawosz^1^, Anna Jung^2^, Grzegorz Gielerak^2^, Henryk Jaremek^3^, Witold Łojkowski^4^, Bartosz Woźniak^4^, Leszek Stobiński^5^, Artur Małolepszy^5^, André Chwalibog^6^*

^1^ Division of Nanobiotechnology, Warsaw University of Life Science, Ciszewskiego 8, 02-786 Warsaw, Poland

^2^ Military Institute of Medicine, Szaserów 128, 04-141 Warsaw, Poland.

^3^ Braster S.A., Cichy Ogród 7, 05-580 Ożarów Mazowiecki, Poland.

^4^ Institute of High Pressure Physics of the Polish Academy of Sciences, Sokołowska 29/37, 01-142 Warsaw, Poland.

^5^ Faculty of Chemical and Process Engineering, Warsaw University of Technology, Waryńskiego 1, 00-645 Warsaw, Poland.

^6^ Department of Veterinary and Animal Sciences, University of Copenhagen, Groennegaardsvej 3, 1870 Frederiksberg, Denmark

*Corresponding author

|  | 1 | 2 | 3 | 4 | 5 | 6 | 7 | 8 | 9 | 10 | 11 | 12 |
| --- | --- | --- | --- | --- | --- | --- | --- | --- | --- | --- | --- | --- |
| A | Pos | Neg | Blank | Blank | TIMP2 | PDGF-BB | sTNF RII | sTNF RI | TNF-β | TNF-α | TGF- β1 | CCL5 |
| B | Pos | Neg | Blank | Blank | TIMP2 | PDGF-BB | sTNF RII | sTNF RI | TNF-β | TNF-α | TGF- β1 | CCL5 |
| C | MIP-1δ | MIP-1β | MIP-1α | CXCL9 | M-CSF | MCP-2 | MCP-1 | CXCL10 | IL-17 | IL-16 | IL-15 | IL-13 |
| D | MIP-1δ | MIP-1β | MIP-1α | CXCL9 | M-CSF | MCP-2 | MCP-1 | CXCL10 | IL-17 | IL-16 | IL-15 | IL-13 |
| E | IL-12 p70 | IL-12 p40 | IL-11 | IL-10 | IL-8 | IL-7 | IL-6sR | IL-6 | IL-4 | IL-3 | IL-2 | IL-1b |
| F | IL-12 p70 | IL-12 p40 | IL-11 | IL-10 | IL-8 | IL-7 | IL-6sR | IL-6 | IL-4 | IL-3 | IL-2 | IL-1b |
| G | IL-1α | CCL1 | INF-γ | ICAM-1 | GM-CSF | GCSF | CCL24 | CCL11 | Neg | Neg | Pos | Pos |
| H | IL-1α | CCL1 | INF-γ | ICAM-1 | GM-CSF | GCSF | CCL24 | CCL11 | Neg | Neg | Pos | Pos |

**Figure S1. Antibody array map.**

Abbreviations: Blank, blank spots without antibodies; CCL1, C-C motif chemokine ligand 1; CCL5, C-C motif chemokine ligand 5; CCL24, C-C motif chemokine ligand 24; CXCL9, C-X-C motif chemokine ligand 9; CXCL10, CCL11 C-X-C motif chemokine ligand 11; GCSF, granulocyte-colony stimulating factor; GM-CSF, granulocyte-macrophage colony-stimulating factor; ICAM-1, intercellular adhesion molecule 1; IL-1α, interleukin 1 alpha; IL-1b, interleukin 1 beta; IL-2, interleukin 2; IL-3, interleukin 3; IL-4, interleukin 4; IL-6, interleukin 6; IL-6sR, soluble interleukin 6 receptor; IL-7, interleukin 7; IL-8, interleukin 8; IL-10, interleukin 10; IL-11, interleukin 11; IL-12 p40, interleukin 12 p40 subunit; IL-12 p70, interleukin 12 p70 subunit; IL-13, interleukin 13; IL-15, interleukin 15; IL-16, interleukin 16; IL-17, interleukin 17; INF-γ, interferon-gamma; MCP-1, monocyte chemoattractant protein 1; MCP-2, monocyte chemoattractant protein 2; M-CSF, macrophage colony-stimulating factor; MIP-1α, macrophage inflammatory protein 1 alpha; MIP-1β, macrophage inflammatory protein 1 beta; MIP-1δ, macrophage inflammatory protein 1 gamma; Neg, negative controls (spots without antibodies printed with antibody dilution buffer); PDGF-BB, platelet-derived growth factor subunit B homodimer; POS, spot with biotin-conjugated IgG; sTNF RI, soluble tumor necrosis factor receptor I; sTNF RII, soluble tumor necrosis factor receptor II; TIMP2, tissue inhibitor of metalloproteinases 2; TGF-β, transforming growth factor beta; TNF-α, tumor necrosis factor alpha; TNF-β, tumor necrosis factor beta;
